# Association of ANGPTL8 and Resistin With Diabetic Nephropathy in Type 2 Diabetes Mellitus

**DOI:** 10.3389/fendo.2021.695750

**Published:** 2021-09-15

**Authors:** Mengni Li, Rongping Fan, Xuemin Peng, Jiaojiao Huang, Huajie Zou, Xuefeng Yu, Yan Yang, Xiaoli Shi, DeLin Ma

**Affiliations:** ^1^Department of Endocrinology, Tongji Hospital, Tongji Medical College, Huazhong University of Science and Technology, Wuhan, China; ^2^Wuhan Branch of National Clinical Research Center for Metabolic Diseases, Wuhan, China

**Keywords:** type 2 diabetes mellitus, ANGPTL-8, resistin, diabetic nephropathy, non-alcoholic fatty liver, predictive effect

## Abstract

**Background:**

Previous studies showed altered angiopoietin-like protein-8 (ANGPTL-8) and resistin circulating levels in type 2 diabetes mellitus (T2DM). Whether or not the alteration in ANGPTL-8 and resistin level can be a predictive maker for increased diabetic nephropathy risk remains unclear.

**Aim:**

To Investigate the possible association of ANGPTL-8 and resistin with DN, and whether this association is affected by NAFLD status.

**Methods:**

A total of 278 T2DM patients were enrolled. Serum levels of ANGPTL8, resistin, BMI, blood pressure, duration of diabetes, glycosylated hemoglobin (HbA1c), fasting blood glucose (FPG), hypersensitive C-reactive protein (hs-CRP), lipid profile, liver, and kidney function tests were assessed. The relationship between DN with ANGPTL8 and resistin was analyzed in the unadjusted and multiple-adjusted regression models.

**Results:**

Serum levels of ANGPTL8 and resistin were significantly higher in DN compared with T2DM subjects without DN (respectively; P <0.001), especially in non-NAFLD populations. ANGPTL8 and resistin showed positive correlation with hs-CRP (respectively; *P*<0.01), and negative correlation with estimated GFR (eGFR) (respectively; *P*=<0.001) but no significant correlation to HOMA-IR(respectively; P>0.05). Analysis showed ANGPTL8 levels were positively associated with resistin but only in T2DM patients with DN(r=0.1867; *P*<0.05), and this significant correlation disappeared in T2DM patients without DN. After adjusting for confounding factors, both ANGPTL8(OR=2.095, 95%CI 1.253-3.502 P=0.005) and resistin (OR=2.499, 95%CI 1.484-4.208 P=0.001) were risk factors for DN. Data in non-NAFLD population increased the relationship between ANGPTL8 (OR=2.713, 95% CI 1.494-4.926 P=0.001), resistin (OR=4.248, 95% CI 2.260-7.987 P<0.001)and DN. The area under the curve (AUC) on receiver operating characteristic (ROC) analysis of the combination of ANGPTL8 and resistin was 0.703, and the specificity was 70.4%. These data were also increased in non-NAFLD population, as the AUC (95%CI) was 0.756, and the specificity was 91.2%.

**Conclusion:**

This study highlights a close association between ANGPTL8, resistin and DN, especially in non-NAFLD populations. These results suggest that ANGPTL-8 and resistin may be risk predictors of DN.

## Introduction

Diabetes is a chronic and progressive disease characterized by metabolic disorders, whose epidemic poses a major global health threat (1). The incidence of diabetes has enormously increased and the global diabetes prevalence in 2019 is estimated to be 9.3% (463 million people), rising to 10.2% (578 million) by 2030 and 10.9% (700 million) by 2045 (2). Type 2 diabetes mellitus (T2DM), which is predominantly due to insulin resistance (IR) and deficiency in insulin secretion (3), accounts for more than 90% of diabetes (2). T2DM patients are prone to occur microvascular and macrovascular complications that cause devastating consequences (4). One microvascular complication, diabetic nephropathy (DN), is the most common cause of end-stage renal disease in developed countries (5). Chronic kidney disease in the setting of DN manifests clinically as albuminuria, reduced glomerular filtration rate (GFR), or both of them (6). Approximately 10% of deaths in type 2 diabetes can be attributed to renal failure (7). Early diagnosis of DN is a crucial component in the management of diabetic nephropathy, therefore timely diagnosis and prompt management of the condition are of paramount importance for effective treatment (8).

ANGPTL8 is a newly identified peptide hormone produced in liver and adipose tissue that play a role in glucose as well as lipid homeostasis (9). Betatrophin is another name given to ANGPTL8, which is also called “hepatocellular carcinoma-associated gene” (TD26), “refeeding induced in fat and liver” (RIFL) (9)and “lipasin” (10). Long-term controversy regarding the role of ANGPTL8 in beta-cell proliferation was resolved by a study that demonstrated ANGPTL8 does not stimulate significant β-cell proliferation (11). However, there are contradictory of findings with respect of serum level of ANGPTL8 in T2DM, some studies reported that ANGPTL8 was increased in T2DM individuals (12, 13) in contrast with a lower concentration of ANGPTL8 reported by Gomez−Ambrosi et al (14). In addition to being related to T2DM, ANGPTL8 is also involved in diabetic complications or other comorbid diseases, such as DN and non-alcoholic fatty liver (NAFLD) (15). Chen et al. found that ANGPTL8 is significantly increased in T2DM patients with different stages of albuminuria (16). Therefore, ANGPTL8 is considered to be involved in the regulation of DN development and more research on its mechanism in T2DM and DN remains to be conducted.

Resistin, originally described as adipose-tissue-specific secretory factor belonging to a cysteine-rich protein family, is almost expressed in white adipocytes and blood cells in mice (17).while in humans, it is mainly found in monocytes/macrophages (18). Due to its production being related to immune system cells and its similar properties to cytokines, resistin was thought to be a possible inflammatory marker in humans (19). In addition to pro-inflammatory effects, resistin also plays a key role in metabolic diseases. Previous studies have shown that resistin has been linked to obesity, diabetes, insulin resistance, DN and NAFLD (20). Gharibeh et al. reported that Serum resistin levels were higher in T2DM patients than the controls and higher in type 2 diabetic obese patients compared with non-diabetic obese subjects (21). A study of 238 patients with T2DM demonstrated that serum levels of resistin were associated with the stage of DN (22). Resistin may also be involved in the regulation of DN development in T2DM.

These serum markers, ANGPTL8 and resistin, may allow earlier diagnosis of DN, but further studies are needed to clarify their predictive value. In addition, there is another factor that required consideration. NAFLD, known as the most common chronic liver disease in the world, is closely related to lipid metabolism (23, 24). The co-exist of NAFLD and T2DM increases the likelihood of the development of complications of diabetes including both microvascular and macrovascular complications (25). ANGPTL8 is produced in liver and it plays an important role in lipid homeostasis (9). Meanwhile, resistin was reported on animal models that liver is its major target (9, 26). Considering both ANGPTL8 and resistin are affected by NAFLD state, whether the above association between ANGPTL8, resisitin with DN is affected by NAFLD state is unclear. Therefore, the present study was performed to investigate the relationship between ANGPTL8, resistin and DN, and to evaluate possible role of ANGPTL8 and resistin in predicting DN in NAFLD state.

## Patients and Methods

### Study Population

A total of 310 T2DM patients from Tongji Hospital in Wuhan, Hubei, China were enrolled between March 2019 and June 2019. Exclusion criteria were as follows: (1) patients with chronic renal disease; (2) any evidence of active infection (e.g. fever or leukocytosis); (3) acute cardiovascular and cerebrovascular events; (4) acute diabetic complications; (5) patients without complete data; (6) patients refused to provide informed consent. Finally, 278 patients were included in our current study. This study was approved by the ethics committee of Tongji hospital (IRB ID : TJ-C20160206). The procedures complied with the provisions of the Declaration of Helsinki. Written informed consent was obtained from all participants.

### Measurements

Blood samples were collected after the participants had fasted overnight and were then centrifuged at 1000g for 15 min at 4°C. Plasma samples were stored at −80°C for subsequent experiments. Fasting plasma glucose (FPG), serum triglyceride (TG), total cholesterol (TC), low-density lipoprotein cholesterol (LDL-C), high-density lipoprotein cholesterol (HDL-C) levels, aspartate transaminase (AST), aminotransferase activity (ALT), renal function including blood urea nitrogen (BUN) serum creatinine (Scr), glomerular filtration rate (eGFR), urine albumin/creatinine ratio (UACR) were measured using a Cobas 8000 auto-analyzer. The hemoglobin (HbA1c) levels were determined using high-performance liquid chromatography (Bio-Rad, Hercules, Calif., USA). Homeostasis model assessment of insulin resistance (HOMA-IR), and homeostasis model assessment of beta cell function (HOMA-β) were also evaluated according to the HOMA calculator. Body mass index (BMI) was calculated using the standard BMI formula: BMI=weight (kg)/height (m)^2^.

The levels of resistin were measured by a Human Quantikine enzyme linked immunosorbent assay (ELISA) kit (R&D Systems Inc., Minneapolis, Minnesota, United States). ANGPTL8 levels were measured by ELISA kit provided by EIAAB (EIAab Science, Wuhan, China; Catalogue No. E11644 h). These measurements were in accordance with the manufacturer’s instructions.

### Definitions

The diagnostic for T2DM meets the American Diabetes Association recommendations (27). The definition of DN was UACR higher than 30 mg/g or eGFR<60 mL/min per 1.73 m^2^ (28). The diagnosis of NAFLD was in line with the Asia–Pacific Working Party. NAFLD was determined by ultrasonography, which was carried out by skilled radiologists. The increase of hepatic-renal echo-intensity ratio was used to evaluate hepatic steatosis. Blood pressure was obtained three times using an automated sphygmomanometer.

### Statistical Analysis

Statistical analyses were conducted using SPSS 26.0 software. P value of <0.05 was considered statistically significant. Continuous variables were described as means ± standard deviation (SD) or medians (interquartile ranges, IQRs) and categorical variables as numerals (percentages). Distribution of the continuous variables was carried out by Kolmogorov-Smirnov Test. The independent Student’s t test for normal distribution and the Mann-Whitney test for asymmetric distribution were used to analyze the differences in continuous variables. The Chi square test was used to analyze categorical variables. The Spearman’s correlation analysis was performed to examine the relationship between ANGPTL8 and resistin with other parameters. Univariate and multivariate logistic regression analyses were used evaluate the associations between ANGPTL8 and resistin respectively with DN. The median values were regarded as the cutoff point when we turned continuous variables into categorical variable. The accuracy of ANGPTL8 as well as resistin as biomarkers for DN was calculated by area under the receiver operating characteristic (ROC) curve.

## Results

### Characteristics of Subjects

A total of 287 T2DM patients were divided into non-DN group and DN group according to the presence of DN. The baseline characteristics of the patients were shown in **Table 1**. Compared with non-DN group, the DN group had higher levels of AST, BUN, Scr, eGFR, UACR and were more likely to have hypertension (P<0.05). No significant difference was observed in age, sex, BMI, HbA1c, FPG, HOMA-IR, HOMA-β, duration of diabetes, TG, TC, LDL-C, HDL-C, ALT and the occurrence of smoking, drinking, NAFLD between the two groups.

**Table 1:** Anthropometric and biochemical characteristics of participants.

Variables	Non-DN (n=159)	DN (n=119)	*P* value
Age (years)	52.08 ± 13.20	55.31 ± 13.94	0.05
Male (%)	104 (65.4)	77 (64.7)	0.903
BMI (kg/m^2^)	24.87 ± 4.20	24.55 ± 3.77	0.427
HbA1c (%)	8.90 ± 2.42	9.39 ± 2.48	0.103
FPG (mmol/L)	8.27 ± 2.99	8.39 ± 3.15	0.749
HOMA-IR	5.21 (2.78,8.26)	6.41 (3.44,11.11)	0.051
HOMA-β	139.50(75.00,233.00)	152.90(69.10,285.30)	0.323
Duration of diabetes (years)	5.00 (1.00,11.00)	6.00 (1.00,12.00)	0.593
TG(mmol/L)	2.31 ± 3.06	2.33 ± 1.81	0.953
TC (mmol/L)	4.15 ± 1.23	4.13 ± 1.16	0.886
LDL-C (mmol/L)	2.48 ± 0.89	2.51 ± 0.93	0.806
HDL-C (mmol/L)	1.06 ± 0.29	1.01 ± 0.29	0.183
AST (U/L)	22.59 ± 15.74	28.14 ± 28.47	**0.043**
ALT (U/L)	30.02 ± 44.03	29.53 ± 34.03	0.920
BUN (mmol/l)	5.93 ± 1.87	7.20 ± 4.78	**0.003**
Scr (μmol/L)	67.58 ± 16.72	104.71 ± 59.70	**<0.001**
eGFR (mL/min/1.73 m^2^)	100.95 ± 16.11	75.00 ± 31.10	**<0.001**
UACR (mg/mmol)	9.53 ± 7.05	797.99 ± 1558.68	**<0.001**
hs-CRP(mmol/l)	8.91 ± 25.42	29.67 ± 50.23	**<0.001**
Smoking (%)	46 (28.9)	93 (33.5)	0.065
Drinking (%)	29 (18.4)	18 (15.1)	0.479
Hypertension (%)	68 (42.8)	70(58.8)	**0.008**
NAFLD (%)	57 (35.8%)	34 (28.6%)	0.201

Data are means ± SD or median (interquartile range). BMI, body mass index; HbA1c, hemoglobin A1c; FPG, fasting plasma glucose concentration; HOMA-IR, homeostasis model assessment of insulin resistance; HOMA-β, homeostasis model assessment of beta cell function; TG, triglyceride; TC, total cholesterol; LDL-C, low-density lipoprotein cholesterol; HDL-C, high density lipoprotein cholesterol; AST, aspartate transaminase; ALT, aminotransferase activity; BUN, blood urea nitrogen; Scr, Serum Creatinine; eGFR, glomerular filtration rate; UACR, urine albumin/creatinine ratio; UAE, urinary albumin excretion; hs-CRP, hypersensitive C-reactive protein;NAFLD, nonalcoholic fatty liver disease. *P* < 0.05 was considered statistically significant. Bold: p < 0.05.

### Elevated Levels of ANGPTL8 and Resistin in T2DM Patients With DN

**Figure 1:**
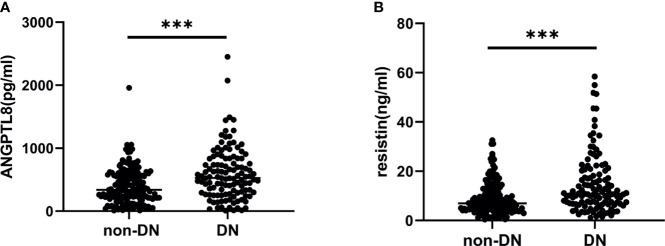
ANGPTL8 and resistin levels in T2DM patients. **(A)**, ANGPTL8 levels were significantly elevated in the DN group. **(B)**, Resistin levels were higher in the DN group. ***p < 0.001 *vs.* non-DN group.

In this study cohort, ANGPTL8 levels were significantly elevated in the DN group compared to the non-DN group (P<0.001) (**Figure 1A**). Similarly, the DN group had higher levels of resistin than the non-DN group (P<0.001) (**Figure 1B**).

### Correlations of ANGPTL8 and Resistin With Clinical Parameters

**Figure 2:**
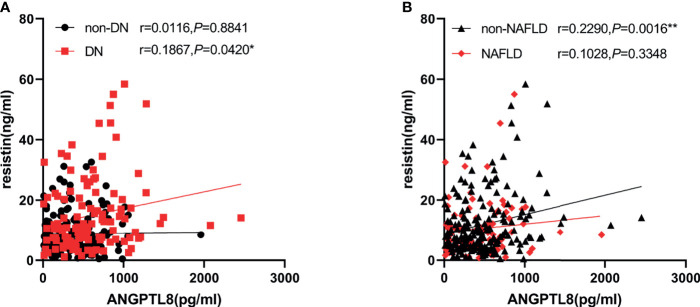
ANGPTL8 was positively associated with resistin in DN in non-NAFLD. **(A)**, ANGPTL8 was positively associated with resistin in DN. **(B)**, ANGPTL8 was positively associated with resistin in non-NAFLD, but not in NAFLD group. *p < 0.05 *vs.* non-DN group. **p < 0.01 *vs.* NAFLD group.

We next investigated the correlation between ANGPTL8 and resistin with a cluster of clinical parameters (**Table 2**). Analysis showed ANGPTL8 levels were positively associated with age (r=0.203 P=0.001), Scr (r=0.160, P=0.009) and UACR (r=0.329 P<0.001). Negative association was observed between ANGPTL8 and eGFR (r=-0.195 P=0.001). Likewise, there was a positive correlation between resistin with BUN (r=0.161 P=0.008), Scr (r=0.278 P<0.001), UACR (R=0.300 P<0.001), and negative correlation between resistin with eGFR was observed (r=-0.289 P<0.001). In addition, we found ANGPTL8 was positively associated with resistin (r=0.1867 P=0.0420) in DN, and this association was not observed in non-DN group. Considering both ANGPTL8 and resistin may be affected by NAFLD state, we introduced NAFLD and non-NAFLD group. ANGPTL8 was positively associated with resistin (r=0.2290 P=0.0016) in non-NAFLD group and this association was not observed in NAFLD group (**Figure 2**).

**Table 2:** Correlations of ANGPTL8 and resistin with anthropometric parameters and biochemical indexes in all patients.

Variables	ANGPTL8			resistin	
	*r*	*P* value		*r*	*P* value
Age (years)	0.203	**0.001**		0.051	0.397
BMI (kg/m^2^)	-0.14	0.816		-0.039	0.513
HbA1c	0.069	0.250		0.059	0.325
FPG	0.079	0.191		0.049	0.414
HOMA-IR	0.079	0.189		0.079	0.188
HOMA-β	0.006	0.922		0.022	0.720
Duration of diabetes	0.068	0.259		0.031	0.610
TG (mmol/L)	-0.022	0.731		0.042	0.489
TC (mmol/L)	-0.085	0.165		-0.093	0.129
LDL-C (mmol/L)	-0.087	0.162		-0.040	0.521
HDL-C (mmol/L)	0.015	0.807		-0.107	0.087
AST (U/L)	0.034	0.581		-0.006	0.917
ALT (U/L)	-0.051	0.404		-0.30	0.628
BUN (mmol/l)	-0.045	0.465		0.161	**0.008**
Scr (μmol/L)	0.160	**0.009**		0.278	**<0.001**
eGFR (mL/min/1.73 m^2^)	-0.195	**0.001**		-0.289	**<0.001**
UACR (mg/mmol)	0.329	**<0.001**		0.300	**<0.001**
hs-CRP(mmol/l)	0.171	**0.004**		0.245	**<0.001**

BMI, body mass index; HbA1c, hemoglobin A1c; FPG, fasting plasma glucose concentration; HOMA-IR, homeostasis model assessment of insulin resistance; HOMA-β, homeostasis model assessment of beta cell function; TG, triglyceride; TC, total cholesterol; LDL-C, low-density lipoprotein cholesterol; HDL-C, high density lipoprotein cholesterol; AST, aspartate transaminase; ALT, aminotransferase activity; BUN, blood urea nitrogen; Scr, Serum Creatinine; eGFR, glomerular filtration rate; UACR, urine albumin/creatinine ratio; UAE, urinary albumin excretion; hs-CRP, hypersensitive C-reactive protein. Bold: p < 0.05.

### ANGPTL8 and Resistin Were Independent Risk Factors for DN

Logistic regression analysis was used to estimate associations between ANGPTL8 and resistin with DN respectively in the study population (**Table 3**). Higher levels of ANGPTL8 (OR=2.306, 95% CI 1.418-3.749, P=0.001) as well as resistin (OR=2.476, 95%CI 1.496-4.098 P<0.001) were risk factors for DN. After adjustment for age, gender and BMI, ANGPTL8(OR=2.179, 95%CI 1.324-3.587 P=0.002) and resistin(OR=2.449, 95%CI 1.474-4.068 P=0.001) were also significantly associated with DN. We then also adjusted other DN risk factors including smoking, drinking, HbA1c and duration of diabetes. The analysis indicated that both ANGPTL8(OR=2.095, 95%CI 1.253-3.502 P=0.005) and resistin (OR=2.499, 95%CI 1.484-4.208 P=0.001) still were risk factors for DN.

**Table 3:** Logistic Regression Analysis of ANGPTL8 and resistin for diabetic nephropathy in all patients.

Models	Variables	Diabetic nephropathy	
		OR (95%CI)	*P*
Model 1	ANGPTL8	2.306(1.418-3.749)	**0.001**
	resistin	2.476(1.496-4.098)	**0.000**
Model 2	ANGPTL8	2.179(1.324-3.587)	**0.002**
	resistin	2.449(1.474-4.068)	**0.001**
Model 3	ANGPTL8	2.095(1.253-3.502)	**0.005**
	resistin	2.499(1.484-4.208)	**0.001**

Model 1: unadjusted.

Model 2: adjusted for age, gender and BMI.

Model 3: adjusted for age, gender, BMI, smoking, drinking, HbA1c and duration of diabetes. Bold: p < 0.05.

Moreover, the ROC curves were used to test the diagnostic accuracy of the ANTPTL8 and resistin for DN (**Figure 4A**). Area under curve (AUC) (95%CI) was 0.646 (0.580 – 0.712) for ANGPTL8, 0.667 (0.602 – 0.731) for resistin and 0.703 (0.641-0.766) for their combination. The optimal cut-off value for detection of DN using ANGPTL8 was 400.45 pg/ml with 67.2% sensitivity and 56.6% specificity. The optimal cut-off value for detection of DN using resistin was 9.28ng/ml with 63.9% sensitivity and 65.4% specificity. The specificity of the combination of ANGPLT8 and resistin was increased at 70.4%.

### The Association of ANGPTL8 and Resistin With DN Was More Obvious in Non-NAFLD Group

**Figure 3:**
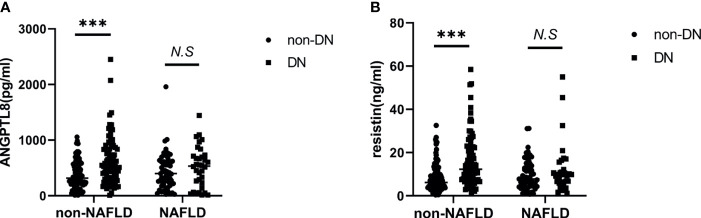
T2DM patients with DN had higher levels of ANGPTL8 and resistin In non-NAFLD population. **(A)**, ANGPTL8 levels were elevated in non-NAFLD population. **(B)**, Resistin levels were elevated in non-NAFLD population. ***p < 0.001 *vs.* NAFLD group. NS, non significance.

**Figure 4:**
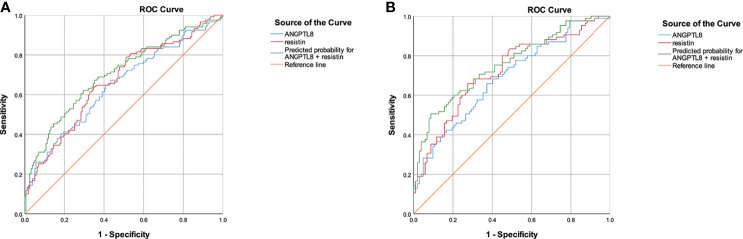
ROC curves for the diagnostic accuracy of the ANTPTL8 and resistin for DN. **(A)**, ROC curves of total population. **(B)**, ROC curves in the non-NAFLD population.

To further explore the effect of NAFLD on the relationship between ANGPTL8 and resistin with DN, T2DM patients were stratified into non-NAFLD population and NAFLD population (**Figure 3**). In the non-NAFLD population, T2DM patients with DN had higher levels of ANGPTL8 and resistin (P<0.001). While in the NAFLD population, the relationship between ANGPTL8 and resistin was lost (P>0.05). Furthermore, logistic regression analysis indicated that there was nearly 3-fold and 4-fold increased risk of DN respectively in higher ANGPTL8 level (OR=2.713, 95% CI 1.494-4.926 P=0.001) and higher resistin level (OR=4.248, 95% CI 2.260-7.987 P<0.001) in the non-NAFLD population, and this association still existed after multiple adjustment (**Table 4**). However, in the NAFLD population, the ANGPTL8 and resistin levels were not associated with DN anymore (P>0.05). Likewise, the ROC curves were performed to identify the value of ANGPTL8 and resistin as biomarkers for DN in the non-NAFLD population (**Figure 4B**). The AUC (95%CI) for ANGPTL8, resistin and their combination were 0.687(0.611-0.763), 0.721(0.647-0.795) and 0.756(0.686-0.825) respectively. The ANGPTL8 cut-off for predicting DN in the non-NAFLD population was higher than 421.47pg/ml, and the sensitivity and specificity were 65.9% and 62.7% respectively. The cut- off value for resistin was 9.84ng/ml with sensitivity and specificity were 65.9% and 72.5% respectively. In addition, the specificity of the combination of ANGPLT8 and resistin was increased at 91.2%.

**Table 4:** Logistic regression analysis of ANGPTL8 and resistin respectively for diabetic nephropathy in the non-NAFLD and NAFLD population.

Models	Variables	Non-NAFLD		NAFLD	
		OR (95%CI)	*P*	OR (95%CI)	*P*
Model 1	ANGPTL8	2.713 (1.494-4.926)	**0.001**	1.742 (0.739-4.104)	0.205
	resistin	4.248 (2.260-7.987)	**0.000**	0.720 (0.281-1.843)	0.493
Model 2	ANGPTL8	2.488 (1.352-4.579)	**0.003**	1.856 (0.756-4.554)	0.177
	resistin	4.149 (2.192-7.855)	**0.000**	0.739 (0.286-1.912)	0.533
Model 3	ANGPTL8	2.437 (1.296-4.579)	**0.006**	2.667 (0.938-7.580)	0.066
	resistin	4.406 (2.275-8.533)	**0.000**	0.758 (0.270-2.123)	0.597

Model 1: unadjusted.

Model 2: adjusted for age, gender and BMI.

Model 3: adjusted for age, gender, BMI, smoking, drinking, HbA1c and duration of diabetes. Bold: p < 0.05.

## Discussion

The present study confirmed that in T2DM patients with DN, circulating levels of ANGPTL8 and resistin were significantly higher than subjects without DN, which was especially obvious in non-NAFLD. In addition, analysis showed ANGPTL8 levels were positively associated with resistin in DN patients. Both of them were independent risk factors for DN, the ROC curve suggested that ANGPTL8 as well as resistin are closely associated with DN, therefore, they may possibly be valuable predictors for DN in T2DM patients. Interestingly, we found that the similar prediction results were more obvious in non-NAFLD group.

Our finding that the serum ANGPTL8 level was elevated in T2DM patients with DN was in agreement with earlier findings (29). An interesting observation in present study was that, unlike other studies (30, 31), ANGPTL8 correlated directly and significantly with hs-CRP, but not with homeostatic model assessment insulin resistance (HOMA-IR), where there was no such significant correlation. ANGPTL8 is a liver-derived hormone that has been suggested may be capable of regulate insulin resistance. Insulin resistance correlates with the development of microalbuminuria in patients with T2DM, for insulin resistance per se contributes to higher salt sensitivity, which closely associates with increase in blood pressure, albuminuria and renal function decline (32, 33).Generally considered, ANGPTL8 may drive the progression of DN through pathways and mechanisms related to insulin resistance. However, our studies seem to indicate that ANGPTL8 could be not responsible for the insulin resistance, which consistent with other different view (34). Apart from this, Guo et al. inversely found that ANGPTL8 alleviates insulin resistance *via* the Akt-GSK3β or Akt-FoxO1 pathway in HepG2 cells (35)and Luo et al. suggested that ANGPTL8 attenuates insulin resistance in diet-induced obese mice (36). These results are controversial, the role of ANGPTL8 in insulin resistance need to elucidate in future studies. Therefore, we speculate that ANGPTL8 may promote the development of DN through inflammatory mechanisms. ANGPTL8 has been proven to positively correlate with hs-CRP, leading to higher inflammatory conditions in thoracic aortic dissection (37). Sinha SK et al (38) reported that elevated serum levels of hs-CRP were associated with, and possibly influenced the development of DN, even for those without DM at baseline. CRP may promote CD32b- NF-κB signaling to mediate renal inflammation and may enhance renal fibrosis in T2DN *via* CD32b-Smad3-mTOR signaling (39). However, these studies failed to clarify how ANGPTL8 affects the development of DN through hsCRP. Larger prognostic studies are needed to validate the cause-effect relationship between ANGPTL8 and deteriorated kidney functions.

The link between resistin and DN in our study was concordant to previous study that mean serum resistin was higher in subjects with advanced nephropathy (22). High levels of resistin can trigger a proinflammatory state by inducing the expression of the proinflammatory cytokines, favoring insulin resistance and enhancing oxidative stress (40). Similar to ANGPTL8, resistin was not significantly correlated with HOMA-IR but significantly correlated with hsCRP. Axelsson et al. declared that resistin associated with both the rate of glomerular filtration and the inflammatory status, but not insulin resistance in chronic kidney disease (DN accounts for 51.35%) (41).Another study on a Japanese population also found that high resistin level was a significant relevant factor in CKD but independent of HOMA-IR (42). Oxidative stress and subclinical inflammation, appear to contribute to the pathogenesis of DN (8). Previous investigations showed that resistin could induce the expression of the proinflammatory cytokines IL-1, IL-6, IL-8, and TNF-α (43, 44), whose synthesis has been observed occurs in several types of renal cells (glomerular, endothelial, tubular and mesangial), as well as in monocytes, macrophages and T cells (45). One particular study concluded that kidney damage evidenced by abnormal renal morphology was directly associated with hyperglycemia-induced oxidative stress in diabetic db/db mice, while oligonol exhibits anti-diabetic effect, reducing glomerular hypertrophy and mesangial matrix expansion caused by diabetes, through down-regulation of circulating resistin and IL-6 levels, demonstrating that kidney damage was able to be protected *via* suppressing inflammatory markers (46). It was further confirmed that the mechanisms by which resistin affects DN development are inflammation and oxidative stress.

ANGPTL8 and resistin were closely associated with DN, and the ROC curve confirmed their relevance. This broadly supported the work of other studies, Yasmine et al (29) reported a possible predictive role of ANGPTL8 in DN and Bruno et al. demonstrated that resistin could predict further renal deterioration eventually leading to end-stage renal disease (ESRD) (47). As both of ANGPTL8 and resistin could contribute to inflammation, we next explored the association between them. Interestingly, our research firstly found that ANGPTL8 levels were positively associated with resistin only in T2DM patients with DN, this significant correlation disappeared in T2DM patients without DN, which encouraged us to investigate whether a combination of different biomarkers could improve the accuracy of predicting DN. Our results found that compared with ANGPTL8 or resistin alone, the AUC combination of ANGPTL8 and resistin was increased. What’s more, the AUC of ROC curve of predicting DN was higher in non-NAFLD group, which demonstrated that their ability to evaluate presence of DN could be enhanced in this part of the population. The significant correlation between ANGPTL8 and resistin in the non-fatty liver group further confirmed this view. Previous study observed that cumulative incidence of DN in patients from group with NAFLD was higher than in group without NAFLD, demonstrating that NAFLD was a risk factor for the development of DN (48). However, our finding was contrary to this finding. We speculated that the association between ANGPTL8, resistin and DN was diminished due to an interference effect of NAFLD on this relationship. For Prior studies noted that ANGPTL8 was significantly increased in mice and humans with NAFLD (49). The precise causal relationship between the elevated ANGPTL8 and NAFLD pathogenesis is not clear. Evidence showed endoplasmic reticulum stress plays important role in the mechanism of ANGPTL8 elevation (15, 49). Meanwhile, it also associated with the development of NAFLD (15, 49). Previous study proved that ANGPTL8 is able to reduce serum triglyceride clearance through the inhibition of lipoprotein lipase(LPL), which hydrolyzes triglycerides, therefore, increased ANGPTL8 leads to triglycerides accumulation (50, 51). On the other hand, the mechanism of association between NAFLD development and resistin elevation is also unclear. Liver is the main target for resistin, which participates in glucose and lipid metabolism (26). There is evidence supporting that resistin probably aggravate pathologic changes in the liver of patients with NAFLD (52). Furthermore, the serum resistin level is positively related to the severity of inflammation and liver fibrosis in NAFLD patients (53). Thereby, both ANGPTL8 and resistin seem to be tightly associated with NAFLD respectively, and in the current study, the NAFLD state might have too much effect on ANGPTL8 and resistin, and severely interfered with the correlation analysis between ANGPTL8, resistin and DN. Accordingly, the relationship between ANGPTL8, resistin and DN in NAFLD state requires further investigation.

Our study also has several limitations. Firstly, our study is a retrospective cross-sectional study, so it’s hard to determine the causal relationship between serum markers(ANGPTL8 and resistin) and DN, which calls for further investigation. In addition, we lacks a healthy control group, those controls may help us study in more detail. Finally, more related inflammatory indexes were not evaluated and compared with ANGPTL8 and resistin.

## Conclusions

Taken together, our results showed that circulating levels of ANGPTL8 and resistin were increased in T2DM with DN, and both of them were independent risk factors for DN. We first found that ANGPTL8 and resistin were positively related, but whether they have a cross mechanism pathway in the progress of T2DM and DN is unclear. Our findings support that ANGPTL8 and resistin could be applied for clinical application as a potential clinical biomarker to predict DN, especially in non-NAFLD population. However, further studies are needed to clarify the mechanism of these biomarkers involved in the pathogenesis of DN.

## Data Availability Statement

The raw data supporting the conclusions of this article will be made available by the authors, without undue reservation.

## Ethics Statement

The studies involving human participants were reviewed and approved by ethics committee of Tongji hospital (IRB ID : TJ-C20160206). Written informed consent for participation was not required for this study in accordance with the national legislation and the institutional requirements.

## Author Contributions

XS, DM, and ML conceived and designed the study and wrote the manuscript. YY, XS, DM, and XY secured the study’s funding. RF, XP, JH, HZ, XY, and YY acquired and analyzed the data. All authors contributed to the article and approved the submitted version.

## Funding

This work was supported by the National Natural Science Foundation of China (Grants 81670754, 81800686 and 81974114) and funds of Jie Chu Jing Ying foundation (Grants 2018076).

## Conflict of Interest

The authors declare that the research was conducted in the absence of any commercial or financial relationships that could be construed as a potential conflict of interest.

## Publisher’s Note

All claims expressed in this article are solely those of the authors and do not necessarily represent those of their affiliated organizations, or those of the publisher, the editors and the reviewers. Any product that may be evaluated in this article, or claim that may be made by its manufacturer, is not guaranteed or endorsed by the publisher.

## References

[1] Lo JC Ljubicic S Leibiger B Kern M Spiegelman BM. Adipsin Is an Adipokine That Improves β Cell Function in Diabetes. Cell (2014) 158:41–53. doi: 10.1016/j.cell.2014.06.005 24995977 PMC4128197

[2] Saeedi P Petersohn I Salpea P Malanda B Karuranga S Unwin N. Global and Regional Diabetes Prevalence Estimates for 2019 and Projections for 2030 and 2045: Results From the International Diabetes Federation Diabetes Atlas. Diabetes Res Clin Pract (2019) 157:107843. doi: 10.1016/j.diabres.2019.107843 31518657

[3] Diabetes AA Association A American PA. Diagnosis and Classification of Diabetes Mellitus. Diabetes Care (2013) 36:S67–74. doi: 10.2337/dc13-S067 PMC3537273 23264425

[4] Chatterjee S Khunti K Davies MJ. Type 2 Diabetes. Lancet (2017) 389:2239–51. doi: 10.1016/S0140-6736(17)30058-2 28190580

[5] Flyvbjerg A. The Role of the Complement System in Diabetic Nephropathy. Nat Rev Nephrol (2017) 13:311–8. doi: 10.1038/nrneph.2017.31 28262777

[6] Afkarian M Zelnick LR Hall YN Heagerty PJ Tuttle K Weiss NS. Clinical Manifestations of Kidney Disease Among US Adults With Diabetes, 1988-2014. Jama (2016) 316:602–10. doi: 10.1001/jama.2016.10924 PMC5444809 27532915

[7] Susan van D Beulens JW Yvonne T van der S Grobbee DE Nealb B. The Global Burden of Diabetes and Its Complications: An Emerging Pandemic. Eur J Cardiovasc Prev Rehabil (2010) 17:s3–8. doi: 10.1097/01.hjr.0000368191.86614.5a 20489418

[8] Tziomalos K Athyros VG. Diabetic Nephropathy: New Risk Factors and Improvements in Diagnosis. Rev Diabetic Studies: RDS (2015) 12:110. doi: 10.1900/RDS.2015.12.110 26676664 PMC5397986

[9] Gang R Ji YK Smas CM. Identification of RIFL, a Novel Adipocyte-Enriched Insulin Target Gene With a Role in Lipid Metabolism. Am J Physiol Endocrinol Metab (2012) 303:E334–51. doi: 10.1152/ajpendo.00084.2012 PMC3423120 22569073

[10] Fu Z Yao F Abou-Samra AB Zhang R. Lipasin, Thermoregulated in Brown Fat, Is a Novel But Atypical Member of the Angiopoietin-Like Protein Family. Biochem Biophys Res Commun (2013) 430:1126–31. doi: 10.1016/j.bbrc.2012.12.025 23261442

[11] Cox AR Barrandon O Cai EP Rios JS Chavez J Bonnyman CW. Resolving Discrepant Findings on ANGPTL8 in β-Cell Proliferation: A Collaborative Approach to Resolving the Betatrophin Controversy. PLoS One (2016) 11:e0159276. doi: 10.1371/journal.pone.0159276 27410263 PMC4943640

[12] Hu H Sun W Yu S Hong X Qian W Tang B. Increased Circulating Levels of Betatrophin in Newly Diagnosed Type 2 Diabetic Patients. Diabetes Care (2014) 37:2718–22. doi: 10.2337/dc14-0602 25024395

[13] Abu-Farha M Al-Khairi I Cherian P Chandy B Sriraman D Alhubail A. Increased ANGPTL3, 4 and ANGPTL8/betatrophin Expression Levels in Obesity and T2D. Lipids Health Dis (2016) 15:1–9. doi: 10.1186/s12944-016-0337-x 27733177 PMC5062897

[14] Gómez-Ambrosi J Pascual E Catalán V Rodríguez A Ramírez B Silva C. Circulating Betatrophin Concentrations Are Decreased in Human Obesity and Type 2 Diabetes. J Clin Endocrinol Metab (2014) 99:E2004–9. doi: 10.1210/jc.2014-1568 25050901

[15] Luo M Peng D. ANGPTL8: An Important Regulator in Metabolic Disorders. Front Endocrinol (2018) 9:169. doi: 10.3389/fendo.2018.00169 PMC5913278 29719529

[16] Chen C-C Susanto H Chuang W-H Liu T-Y Wang C-H. Higher Serum Betatrophin Level in Type 2 Diabetes Subjects Is Associated With Urinary Albumin Excretion and Renal Function. Cardiovasc Diabetol (2016) 15:1–9. doi: 10.1186/s12933-015-0326-9 26739836 PMC4704426

[17] Steppan CM Brown EJ Wright CM Bhat S Banerjee RR Dai CY. A Family of Tissue-Specific Resistin-Like Molecules. Proc Natl Acad Sci (2001) 98:502–6. doi: 10.1073/pnas.98.2.502 PMC14616 11209052

[18] Yang R-Z Huang Q Xu A McLenithan JC Eison JA Shuldiner AR. Comparative Studies of Resistin Expression and Phylogenomics in Human and Mouse. Biochem Biophys Res Commun (2003) 310:927–35. doi: 10.1016/j.bbrc.2003.09.093 14550293

[19] Bokarewa M Nagaev I Dahlberg L Smith U Tarkowski A. Resistin, an Adipokine With Potent Proinflammatory Properties. J Immunol (2005) 174:5789–95. doi: 10.4049/jimmunol.174.9.5789 15843582

[20] Jamaluddin MS Weakley SM Yao Q Chen C. Resistin: Functional Roles and Therapeutic Considerations for Cardiovascular Disease. Br J Pharmacol (2012) 165:622–32. doi: 10.1111/j.1476-5381.2011.01369.x PMC3315035 21545576

[21] Gharibeh M Al Tawallbeh G Abboud M Radaideh A Alhader A Khabour O. Correlation of Plasma Resistin With Obesity and Insulin Resistance in Type 2 Diabetic Patients. Diabetes Metab (2010) 36:443–9. doi: 10.1016/j.diabet.2010.05.003 20739208

[22] Osawa H Ochi M Kato K Yamauchi J Nishida W Takata Y. Serum Resistin is Associated With the Severity of Microangiopathies in Type 2 Diabetes. Biochem Biophys Res Commun (2007) 355:342–6. doi: 10.1016/j.bbrc.2007.01.144 17303077

[23] Barchetta I Cimini FA Chiappetta C Bertoccini L Ceccarelli V Capoccia D. Relationship Between Hepatic and Systemic Angiopoietin-Like 3, Hepatic Vitamin D Receptor Expression and NAFLD in Obesity. Liver Int (2020) 40:2139–47. doi: 10.1111/liv.14554 32510837

[24] Younossi ZM. Non-Alcoholic Fatty Liver Disease – A Global Public Health Perspective - ScienceDirect. J Hepatol (2019) 70:531–44. doi: 10.1016/j.jhep.2018.10.033 30414863

[25] Hazlehurst JM Woods C Marjot T Cobbold JF Tomlinson JW. Non-Alcoholic Fatty Liver Disease and Diabetes. Metabolism (2016) 65:1096–108. doi: 10.1016/j.metabol.2016.01.001 PMC4943559 26856933

[26] Colica C Abenavoli L. Resistin Levels in Non-Alcoholic Fatty Liver Disease Pathogenesis. J Trans Internal Med (2018) 6:52–3. doi: 10.2478/jtim-2018-0011 PMC5874489 29607306

[27] Association AD. 2. Classification and Diagnosis of Diabetes: Standards of Medical Care in Diabetes—2020. Diabetes Care (2020) 43:S14–31. doi: 10.2337/dc20-S002 31862745

[28] Association AD. 11. Microvascular Complications and Foot Care: Standards of Medical Care in Diabetes– 2020. Diabetes Care (2020) 43:S135–51. doi: 10.2337/dc20-S011 31862754

[29] Issa YA Abd ElHafeez SS Amin NG. The Potential Role of Angiopoietin-Like Protein-8 in Type 2 Diabetes Mellitus: A Possibility for Predictive Diagnosis and Targeted Preventive Measures? EPMA J (2019) 10:239–48. doi: 10.1007/s13167-019-00180-3 PMC6695457 31462941

[30] Chen X Lu P He W Zhang J Liu L Yang Y. Circulating Betatrophin Levels Are Increased in Patients With Type 2 Diabetes and Associated With Insulin Resistance. J Clin Endocrinol Metab (2015) 100:E96. doi: 10.1210/jc.2014-2300 25303484

[31] Pu D Li L Yin J Liu R Yang G Liao Y. Circulating ANGPTL8 Is Associated With the Presence of Metabolic Syndrome and Insulin Resistance in Polycystic Ovary Syndrome Young Women. Mediators Inflamm (2019) 2019:10. doi: 10.1155/2019/6321427 PMC6620840 31346314

[32] Trevisan R Bruttomesso D Vedovato M Brocco S Pianta A Mazzon C. Enhanced Responsiveness of Blood Pressure to Sodium Intake and to Angiotensin II Is Associated With Insulin Resistance in IDDM Patients With Mcroalbuminuria. Diabetes (1998) 47:1347. doi: 10.2337/diab.47.8.1347 9703338

[33] Vedovato M Lepore G Coracina A Dodesini A Jori E Tiengo A. Effect of Sodium Intake on Blood Pressure and Albuminuria in Type 2 Diabetic Patients: The Role of Insulin Resistance. Diabetologia (2004) 47:300–3. doi: 10.1007/s00125-003-1303-5 14704836

[34] Abu-Farha M Abubaker J Al-Khairi I Cherian P Elkum N. Higher Plasma Betatrophin/ANGPTL8 Level in Type 2 Diabetes Subjects Does Not Correlate With Blood Glucose or Insulin Resistance. Sci Rep (2015) 5:10949. doi: 10.1038/srep10949 26077345 PMC4650613

[35] Guo XR Wang XL Chen Y Yuan YH Chen YM Ding Y. ANGPTL8/betatrophin Alleviates Insulin Resistance via the Akt-Gsk3β or Akt-FoxO1 Pathway in HepG2 Cells. Exp Cell Res (2016) 345:158–67. doi: 10.1016/j.yexcr.2015.09.012 26387753

[36] Luo D Chen X Yang W Ran W Wen Z. Angiopoietin-Like 8 Improves Insulin Resistance and Attenuates Adipose Tissue Inflammation in Diet-Induced Obese Mice. Exp Clin Endocrinol Diabetes (2020) 128:290–6. doi: 10.1055/a-0725-7897 30257264

[37] Yang Y Jiao X Li L Hu C Zhang X Pan L. Increased Circulating Angiopoietin-Like Protein 8 Levels Are Associated With Thoracic Aortic Dissection and Higher Inflammatory Conditions. Cardiovasc Drugs Ther (2020) 34:65–77. doi: 10.1007/s10557-019-06924-7 32034642 PMC7093348

[38] Sinha SK Nicholas SB Sung JH Correa A Rajavashisth TB Norris KC. Hs-CRP Is Associated With Incident Diabetic Nephropathy: Findings From the Jackson Heart Study. Diabetes Care (2019) 42:2083–9. doi: 10.2337/dc18-2563 PMC6804609 31511234

[39] You Y-K Huang X-R Chen H-Y Lyu X-F Liu H-F Lan HY. C-Reactive Protein Promotes Diabetic Kidney Disease in Db/Db Mice via the CD32b-Smad3-mTOR Signaling Pathway. Sci Rep (2016) 6:1–14. doi: 10.1038/srep26740 27221338 PMC4879671

[40] Santilli F Liani R Di Fulvio P Formoso G Simeone P Tripaldi R. Increased Circulating Resistin Is Associated With Insulin Resistance, Oxidative Stress and Platelet Activation in Type 2 Diabetes Mellitus. Thromb Haemostasis (2016) 116:1089–99. doi: 10.1160/TH16-06-0471 27709225

[41] Axelsson J Bergsten A Qureshi A Heimbürger O Barany P Lönnqvist F. Elevated Resistin Levels in Chronic Kidney Disease Are Associated With Decreased Glomerular Filtration Rate and Inflammation, But Not With Insulin Resistance. Kidney Int (2006) 69:596–604. doi: 10.1038/sj.ki.5000089 16395259

[42] Kawamura R Doi Y Osawa H Ninomiya T Hata J Yonemoto K. Circulating Resistin Is Increased With Decreasing Renal Function in a General Japanese Population: The Hisayama Study. Nephrol Dialysis Transplant (2010) 25:3236–40. doi: 10.1093/ndt/gfq155 20339098

[43] Filková M Haluzík M Gay S Šenolt L. The Role of Resistin as a Regulator of Inflammation: Implications for Various Human Pathologies. Clin Immunol (2009) 133:157–70. doi: 10.1016/j.clim.2009.07.013 19740705

[44] Silswal N Singh AK Aruna B Mukhopadhyay S Ghosh S Ehtesham NZ. Human Resistin Stimulates the Pro-Inflammatory Cytokines TNF-α and IL-12 in Macrophages by NF-κb-Dependent Pathway. Biochem Biophys Res Commun (2005) 334:1092–101. doi: 10.1016/j.bbrc.2005.06.202 16039994

[45] Navarro-González JF Mora-Fernández C De Fuentes MM García-Pérez J. Inflammatory Molecules and Pathways in the Pathogenesis of Diabetic Nephropathy. Nat Rev Nephrol (2011) 7:327–40. doi: 10.1038/nrneph.2011.51 21537349

[46] Liu H-W Wei C-C Chang S-J. Low-Molecular-Weight Polyphenols Protect Kidney Damage Through Suppressing NF-κb and Modulating Mitochondrial Biogenesis in Diabetic Db/Db Mice. Food Funct (2016) 7:1941–9. doi: 10.1039/C6FO00078A 26960417

[47] Bonito B Silva AP Rato F Santos N Neves PL. Resistin as a Predictor of Cardiovascular Hospital Admissions and Renal Deterioration in Diabetic Patients With Chronic Kidney Disease. J Diabetes Its Complications (2019) 33:107422. doi: 10.1016/j.jdiacomp.2019.107422 31484628

[48] Jia G Di F Wang Q Shao J Gao L Wang L. Non-Alcoholic Fatty Liver Disease Is a Risk Factor for the Development of Diabetic Nephropathy in Patients With Type 2 Diabetes Mellitus. PLoS One (2015) 10:e0142808. doi: 10.1371/journal.pone.0142808 26566287 PMC4643958

[49] Lee YH Lee SG Lee CJ Kim SH Song YM Yoon MR. Association Between Betatrophin/ANGPTL8 and Non-Alcoholic Fatty Liver Disease: Animal and Human Studies. Sci Rep (2016) 6:24013. doi: 10.1038/srep24013 27045862 PMC4820743

[50] Zhang R Abou-Samra AB. Emerging Roles of Lipasin as a Critical Lipid Regulator. Biochem Biophys Res Commun (2013) 432:401–5. doi: 10.1016/j.bbrc.2013.01.129 23415864

[51] Mele C Crinò A Fintini D Mai S Marzullo P. Angiopoietin-Like 8 (ANGPTL8) as a Potential Predictor of NAFLD in Paediatric Patients With Prader-Willi Syndrome. J Endocrinol Invest (2020) 44:3. doi: 10.1007/s40618-020-01444-w PMC8195791 33067796

[52] Gierej P Gierej B Kalinowski P Wróblewski T Paluszkiewicz R Kobryń K. Expression of Resistin in the Liver of Patients With Non-Alcoholic Fatty Liver Disease. Polish J Pathol (2017) 68:225. doi: 10.5114/pjp.2017.67583 29363914

[53] Garcia CC Piotrkowski B Baz P Poncino D Benavides J Colombato L. A Decreased Response to Resistin in Mononuclear Leukocytes Contributes to Oxidative Stress in Nonalcoholic Fatty Liver Disease. Digestive Dis Sci (2021) 43:1–11. doi: 10.1007/s10620-021-07105-z 34156590

